# The socio-economic shield limits Lassa virus spillover in urban West Africa

**DOI:** 10.1017/S0950268826101824

**Published:** 2026-06-24

**Authors:** David Simons

**Affiliations:** Department of Anthropology, Pennsylvania State University, USA

**Keywords:** joint species distribution model, Lassa virus, *Mastomys natalensis*, seroreversion, spillover, underreporting, urbanization

## Abstract

Spatial risk models for Lassa fever (LF) generally predict the primary reservoir, *Mastomys natalensis*, is restricted to rural landscapes. This study integrates multispecies biotic interactions and anthropogenic land-use into a high-resolution framework to evaluate LF’s urban potential. I implemented an integrated multispecies occupancy model to reconstruct the reservoir’s realized niche, accounting for sampling bias and invasive rodent competitors. A socio-economic filter, proxied by night-time lights, was introduced to model the dampening effect of urban infrastructure on spillover. Annual infections were estimated using a demographic compartmental model incorporating empirical seroreversion rates. Results indicate high biological hazard across the peri-urban fringes of major West African cities. However, an infrastructure-driven socio-economic shield decouples this hazard from human incidence in dense urban cores. Accounting for spatial shielding and antibody waning yields an estimated 2.6 million annual Lassa virus infections. Comparing predictions to clinical data reveals substantial surveillance gaps, identifying highly suitable silent districts in Nigeria, Benin, and Togo with zero reported cases. LF possesses the biological potential to become a peri-urban disease; addressing these surveillance gaps at the peri-urban interface is a critical public health priority.

## Key findings

- Accounting for species interactions reveals that the LF reservoir (*M. natalensis*) exhibits high ecological tolerance for peri-urban and human-modified landscapes.
- Urban infrastructure acts as a socio-economic shield, decoupling biological hazard from realized human spillover in densely populated city centres.
- Adjusting for empirically modelled antibody waning and urban shielding yields an estimated regional burden of 2.6 million annual LASV infections.
- Spatial validation identifies numerous silent districts across West Africa, revealing surveillance gaps rather than an absence of biological hazard.

## Introduction

The rapid expansion of West African cities simultaneously amplifies human-commensal contact and introduces infrastructural barriers to zoonotic transmission. Forecasting the trajectory of viral haemorrhagic fevers in the Anthropocene requires understanding how human activities (e.g. housing construction and land-use change) shape local rodent dynamics before scaling to regional transmission patterns. Lassa fever (LF), caused by *Mammarenavirus lassaense* (Lassa virus (LASV); Arenaviridae), represents an ideal system to test these dynamics. Its primary reservoir, the generalist commensal *Mastomys natalensis*, is actively being reshaped by these rapid anthropogenic shifts [1].

LF is a significant public health concern across West Africa, yet accurate estimation of the true disease burden remains elusive. This uncertainty is driven by high rates of asymptomatic infection [2] compounded by epidemiological opacity; under-detection is not solely a logistical failure but is shaped by social inequalities, diagnostic capacity, and the spatial distribution of healthcare infrastructure [3–5]. Because passive clinical surveillance primarily captures severe outbreaks, it frequently fails to resolve the underlying geography of endemic transmission.

Robust, spatially explicit risk maps are therefore essential for identifying true biological hazard and guiding public health interventions, including the deployment of future vaccines [6, 7]. Foundational forecasting efforts have successfully utilized mechanistic models to predict environmental suitability for the host ( $D_{M}$ ) and the virus ( $D_{L}$ ), calibrating the composite hazard ( $D_{X}=D_{M}\times D_{L}$ ) against human seroprevalence data [8]. Parallel macroecological approaches have similarly linked climatic suitability directly to the incidence of clinical cases [9]. While these models provided informative regional baselines, their primary reliance on abiotic predictors led to the predicted climatic exclusion of the reservoir from highly modified urban landscapes. As spatial epidemiology increasingly integrates multidisciplinary, cross-scale frameworks [10, 11], relying solely on abiotic envelopes risks underestimating zoonotic hazard within rapidly expanding urban centres [12].

In West Africa’s heterogeneous landscapes, the reservoir’s realized niche is dictated by ecological plasticity and multispecies biotic interactions, rather than climate alone. Recent longitudinal studies demonstrate that *M. natalensis* occupancy increases along a gradient from forested areas to agricultural and village habitats, exhibiting a high degree of synanthropic tolerance [1, 13]. However, in high-density urban environments, this native reservoir faces competitive pressure and potential displacement by invasive commensals, specifically the Black Rat (*Rattus rattus*) and House Mouse (*Mus musculus*) [14–16]. By neglecting these complex community dynamics, previous models have potentially mischaracterized the zoonotic hazard at the urban-bushland interface, creating an urban blind spot in risk forecasting.

Furthermore, spillover is not solely a function of reservoir density, but of interface permeability [17, 18]. In rural landscapes, porous housing materials create a high-contact interface [19–21]. Previous epidemiological frameworks, trained heavily on rural data, have projected these high-contact dynamics into dense cities. In reality, urbanization introduces structural barriers including improved housing, distinct agricultural zoning, and collective refuse management that act to decouple human exposure from rodent density [22]. Models that fail to account for this non-linear ‘socio-economic shield’ risk systematically overestimating spillover in urban centres, potentially directing surveillance resources away from the peri-urban fringes where transmission intensity is highest.

To address this gap, I re-evaluated the spatial hazard of LASV spillover using an integrated multispecies occupancy model (IMSOM) [23, 24]. This framework explicitly quantifies co-occurrence dynamics between *M. natalensis* and the wider rodent community, deriving a refined reservoir layer ( $D_{M}$ ) that accounts for the biotic pressure exerted by competitors.

To resolve the substantial uncertainty surrounding the magnitude of human infection, I integrated recent longitudinal evidence on LASV seroreversion rates to constrain the annual force of infection [25]. Finally, these refined estimates were validated against subnational clinical case reports to quantify the discrepancy between biological hazard and passive surveillance, spatially identifying silent districts to guide the prioritization of future LF control.

## Methods

### Data assembly and environmental predictors

#### Host community and spatial framework

This study focused on the endemic zone of the A-I clade of *M. natalensis* in West Africa, evaluated across a $0.05^{\circ }\times 0.05^{\circ }$ spatial grid. Because the physiological and ecological parameters underlying this model are calibrated to the A-I lineage, caution must be exercised in extrapolating these functional responses, specifically synanthropic tolerance and competitive displacement, to the other lineages without regional recalibration. To characterize the host community, a comprehensive dataset of small mammal occurrences (1972–2022) was compiled from curated trapping databases, targeted literature extraction, and opportunistic records [26–28]. The dataset included eight epidemiologically and ecologically relevant species: the primary reservoir (*M. natalensis*), sympatric native species, and invasive commensals (*R. rattus*, *M. musculus*).

#### Environmental covariates

I reconstructed the environmental predictor stack utilized in previous forecasting efforts, updating the temporal window to 2001–2025 [8]. Predictors captured climate, topography, seasonal environmental stability, and anthropogenic land-use (details in Supplementary Materials). To prevent multicollinearity and ensure ecological parsimony, a final subset of predictors was selected based on variance inflation factors (VIF < 3) and a priori biological hypotheses. This subset included indices of temperature, precipitation, elevation, agricultural density, and a quadratic term for log-transformed human population density to capture non-linear responses to urbanization.

### Ecological modelling: The realized host niche ( $D_{M}$ )

To estimate the spatial hazard of LASV, I first refined the predicted distribution of the reservoir host ( $D_{M}$ ). Previous models relied on abiotic climatic envelopes. However, to account for the competitive exclusion of *M. natalensis* by invasive rodents in urban environments, I implemented an IMSOM using the spOccupancy package in R [23, 29].

Unlike single-species approaches, the IMSOM explicitly models residual correlations between species via latent factor analysis, leveraging community-level data pooling to stabilize detection estimates for unstructured opportunistic records. This joint framework is essential for the subsequent pathogen modelling; it concurrently generates the predicted spatial distributions of invasive competitors (*R. rattus*, *M. musculus*), which is a prerequisite for testing the biotic constraints on viral prevalence. While random background pseudo-absences were generated to characterize available environmental space, spatial sampling bias was controlled for within the model’s observation process, which parameterized detection probability as a function of data source and survey effort (Supplementary Materials).

From the fitted model, I extracted the mean posterior occupancy probability for *M. natalensis* to serve as the refined reservoir hazard layer ( $D_{M}$ ), masked to its International Union for the Conservation of Nature (IUCN) extant range to prevent biogeographical extrapolation. I concurrently extracted the predicted occupancy surfaces for the invasive competitors *R. rattus* and *M. musculus.* Given their low observed rates of active LASV infection and unproven competence for onward transmission, these invasive species were treated primarily as ecological constraints on the reservoir, rather than direct sources of zoonotic hazard. Their occupancy layers were subsequently integrated as biotic covariates in the pathogen model.

### Spillover estimation and epidemiological constraints

#### Modelling the biotic and socio-economic filters

The composite ecological hazard ( $D_{X}$ ) was calculated as the product of the reservoir distribution ( $D_{M}$ ) and pathogen prevalence ( $D_{L}$ ). To construct $D_{L}$ , I trained a boosted regression tree (BRT) on 73 testing sites where *M. natalensis* was assayed for LASV. To explicitly account for the hypothesized dilution effect, predicted occupancies of invasive rodents (*R. rattus*, *M. musculus*) from the IMSOM were included as biotic covariates. To address spatial circularity, any rodent testing site within 5 km of a human serosurvey used for final calibration was excluded from the training set.

Unlike previous models, I explicitly introduced a socio-economic filter using night-time lights (NTL) to proxy electrification and housing quality, hypothesizing that urban infrastructure acts as a physical barrier to rodent contact. Because empirical community-based serosurveys are entirely absent from high-density city centres [30], correlative models risk extrapolating high rural contact rates into novel urban environments. To prevent this ecological fallacy, I augmented the calibration dataset with five synthetic absence anchors ( n=500 , 0% seroprevalence) representing hyperurban commercial centres (e.g. Lagos Island). These act as informative Bayesian priors reflecting the historical absence of autochthonous transmission in these zones, constraining the model to estimate the suppressive effect of extreme urbanization (NTL > 20).

#### Transmission dynamics and incidence estimation

The composite ecological hazard ( $D_{X}$ ) was calibrated against 94 historical human serosurveys using a quasi-binomial Generalized Linear Model (GLM). To convert predicted equilibrium seroprevalence ( $\Omega^{*}$ ) into the annual incidence of new infections, I utilized a steady-state demographic Susceptible-Infectious-Recovered-Susceptible (SIRS) compartmental model [8].

At endemic steady-state, the incidence of new infections must balance the total outflow of individuals recovering and dying (full system of ordinary differential equations provided in Supplementary Materials). By solving this system at equilibrium, the total annual incidence of new infections within a given spatial cell can be derived directly from the observed seropositive fraction ( $\Omega^{*}$ ) and the total population ( N ):

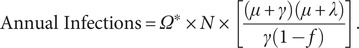



This formulation addresses the demographic reality that a fraction of infected individuals die before seroconverting, and that surviving seropositive individuals are continuously removed via natural demographic turnover ( μ ).

To capture regional demographic heterogeneity, the background mortality rate ( μ ) was treated as a spatially varying covariate, calculated as the reciprocal of country-specific life expectancies. These 2023 estimates were accessed from the World Bank data repository using the WDI package (ranging from 54 years in Nigeria to 69 years in Senegal) [31]. The recovery rate ( γ ) was set to $12\;{\text{year}}^{-1}$ , approximating a one-month infectious and convalescent period [25]. The infection fatality rate (IFR) ( f ) was conservatively set to 2% (0.02) [2].

Uncertainty surrounding the total burden was constrained by evaluating a sensitivity range for the rate of antibody waning ( λ ). Relying on longitudinal serological evidence, I calculated the primary incidence burden, assuming an annual seroreversion rate of 3% ( λ=0.03 ), with sensitivity bounds between lifelong immunity ( λ=0 ) and rapid loss ( λ=0.064 ). Total annual infections per $0.05^{\circ }$ pixel were calculated by integrating these parameters with the WorldPop 2020 human population matrix.

#### Validation and urban gradient analysis

To assess epidemiological accuracy, I compared predicted annual infections against aggregated clinical case reports at subnational (Admin-2) and national scales [32]. I distinguish between total biological transmission events (predicted infections) and the subset of symptomatic, health-seeking individuals (reported cases). Rather than treating discrepancies purely as model error, spatial divergence was utilized to identify silent districts, areas where environmental suitability implies high transmission despite zero reported cases.

Second, to validate the ecological realism of the model within highly modified environments, I formally tested the ‘Urban Paradox’, the hypothesis that infrastructure creates a transmission blind spot in urban cores despite high biological hazard. I conducted a spatially explicit gradient analysis along 50 km radial transects for 104 stratified West African settlements. By extracting mean values for ecological hazard ( $D_{X}$ ), the socio-economic shield (NTL), and realized incidence across these transects, I quantified the spatial decoupling of biological threat and human exposure across different urban typologies (full extraction protocols and statistical methods detailed in Supplementary Materials).

## Results

### Model performance and the cryptic reservoir niche

**Figure 1.**
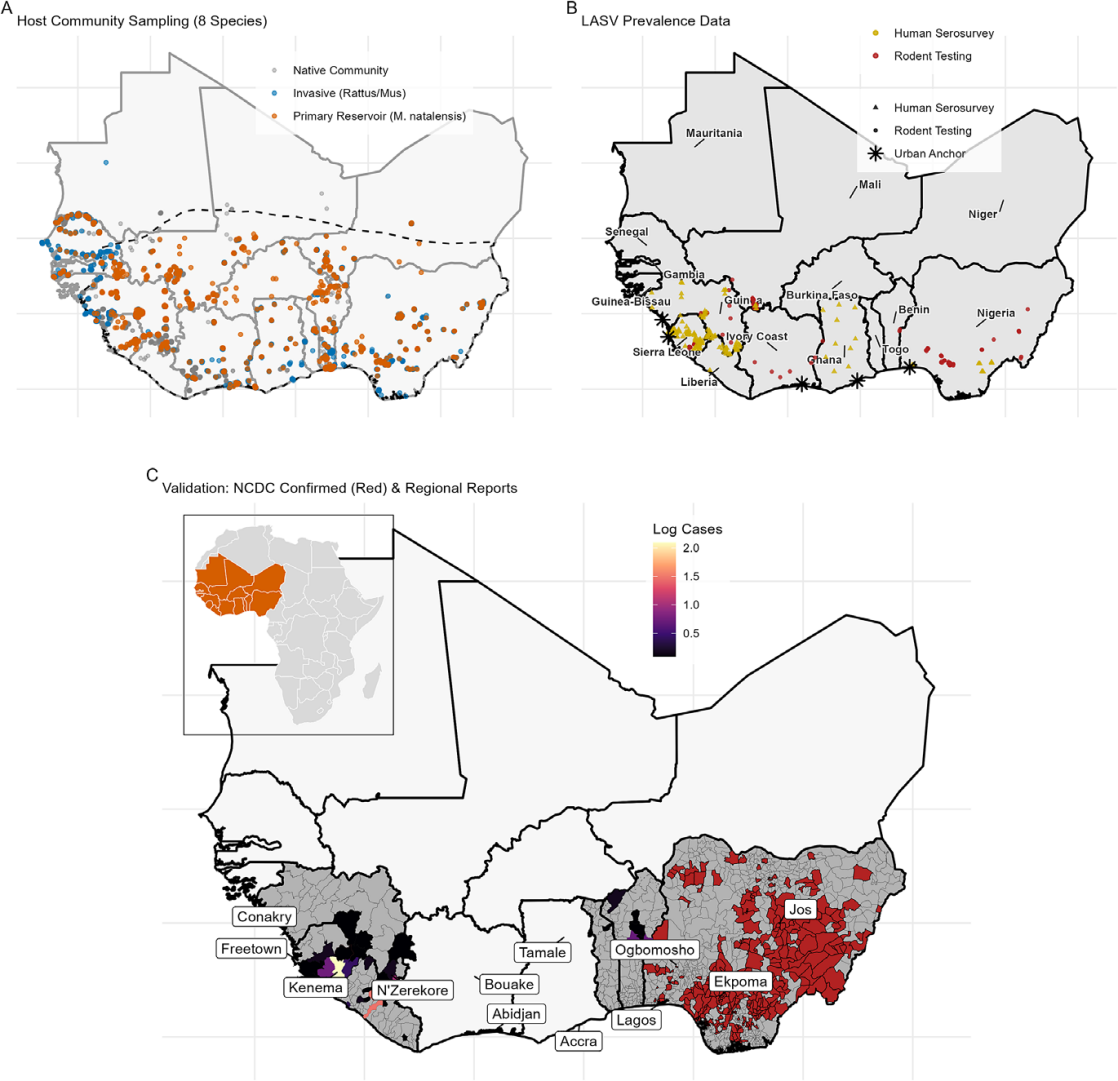
Spatial distribution of biological and epidemiological data. (a) Small mammal trapping locations used to train the IMSOM, stratified by ecological guild: the primary reservoir *M. natalensis* (orange), invasive competitors *R. rattus*/*M. musculus* (blue), and the wider native community (grey). The dashed black line represents the IUCN range of *M. natalensis* within West Africa. (b) LASV prevalence data points. Red circles indicate rodent testing sites (polymerase chain reaction (PCR)/serology); gold triangles indicate human serosurveys used for calibration; black asterisks indicate synthetic urban absence anchors added to constrain model predictions in high-density city centres. (c) Reported cases resolved to administrative level two areas. Red Local Government Areas (LGAs) in Nigeria indicate those that have reported at least one confirmed case between 2018 and 2025 (obtained from weekly situation reports produced by the NCDC). Outside of Nigeria, administrative level two areas are coloured by the number of cases (log transformed) reported in the period 2012–2022. Labels refer to the focal cities for the spatially explicit gradient analyses. The inset map shows the extent of the study area within Africa. Figure 1. long description.

Current understanding of the LASV reservoir niche is constrained by severe sampling bias. Analysis of 4,908 unique rodent trapping locations revealed that 67% of systematic sampling effort (and 95% of opportunistic recording) has been concentrated in rural settings, leaving substantial spatial sampling gaps in the rapidly urbanizing coastal corridors of West Africa (Supplementary Table 1 and Figure 1). However, of the 228 systematic surveys successfully conducted in urban settings, 88.6% reported *M. natalensis* presence, suggesting the reservoir routinely exploits these environments.

To formally test whether anthropogenic factors drive reservoir distribution beyond standard climatic envelopes, I compared a baseline climate-only occupancy model (analogous to previous forecasting efforts) against a model incorporating human population density and land-use. The inclusion of anthropogenic predictors substantially improved model fit ( ΔWAIC=70.83 ), confirming that the reservoir’s spatial limits cannot be defined by climate alone.

**Figure 2.**
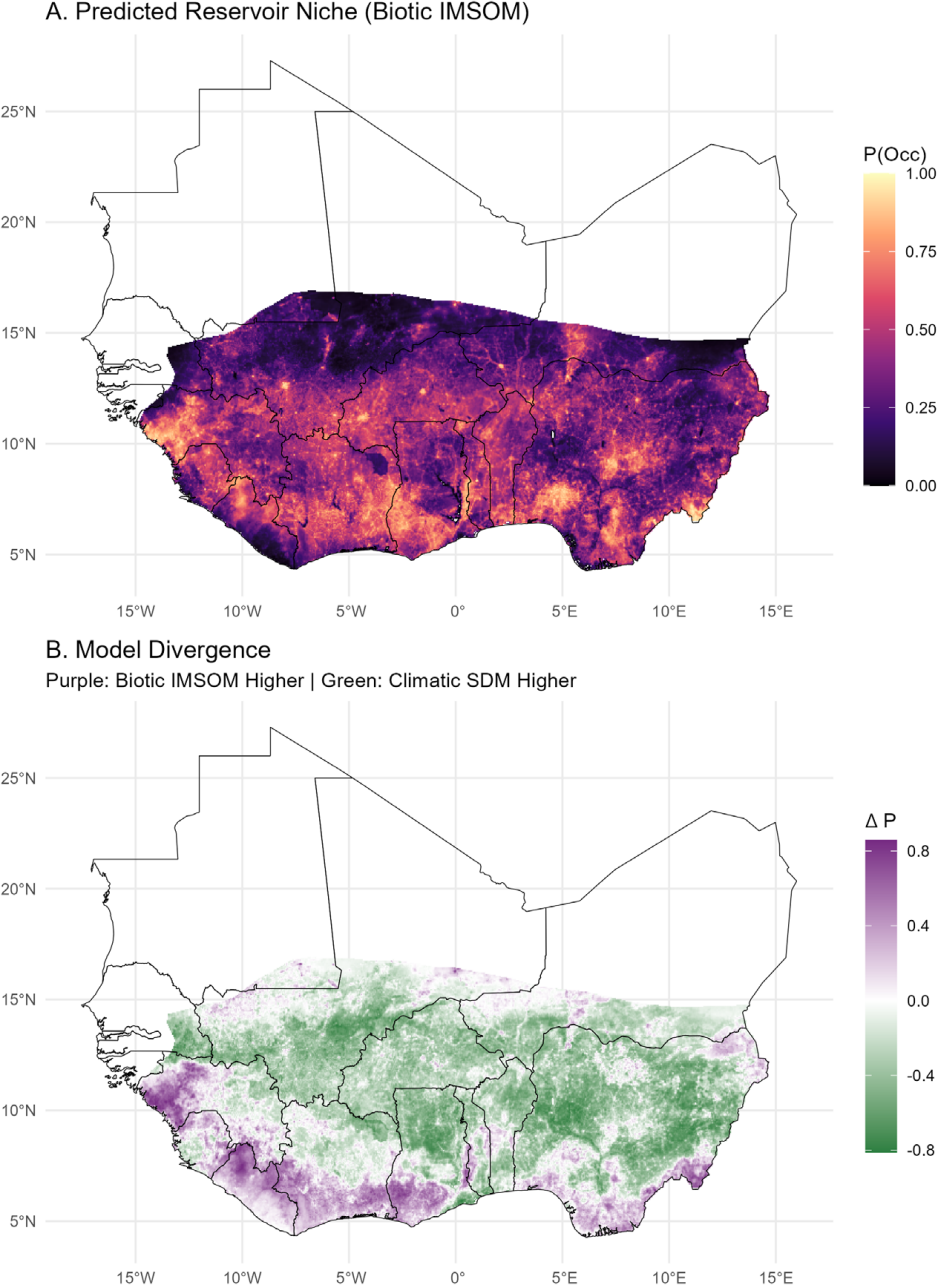
Predicted reservoir niche **(** $D_{M}$ **)**. (a) IMSOM predicted occupancy probability for *M. natalensis.* (b) Difference map highlighting spatial divergence from previous climatic models, with positive values indicating higher predicted suitability in the IMSOM, particularly in urban zones. Figure 2. long description.

To reconstruct this realized niche while accounting for species interactions, I utilized the IMSOM. Previous foundational models, relying strictly on abiotic predictors, explicitly predicted the reservoir to be less prevalent along the heavily modified coastal corridors of southern Nigeria and West Africa [8]. The biotically informed IMSOM corrects this urban blind spot, predicting high occupancy probabilities ( P>0.7 ) across peri-urban and urban zones in these exact coastal regions (Figure 2).

This divergence is driven by the host’s empirically derived functional response to anthropogenic pressure. The IMSOM reveals a strong, positive association between *M. natalensis* occurrence and human population density (posterior mean β=1.14 , 95% credible interval (CrI): 0.75–1.57). This synanthropic tolerance directly contrasts with native forest-specialist rodents like *Malacomys edwardsi*, which exhibited negative associations with anthropogenic disturbance (functional response coefficients for all species are shown in Supplementary Figure 1).

**Figure 3.**
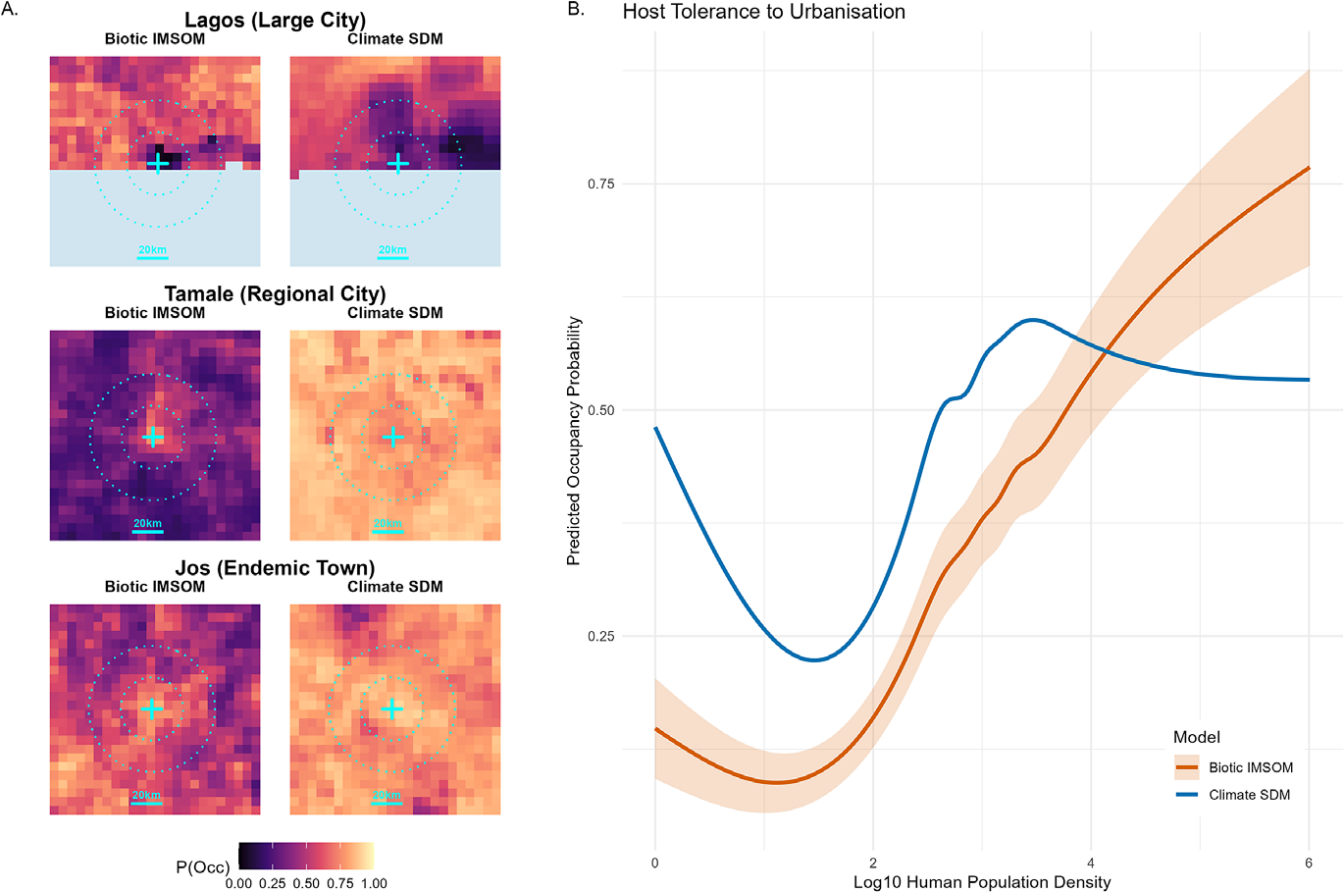
Mechanisms of urban tolerance. (a) Urban zooms showing reservoir persistence in peri-urban fringes for three example locations (Lagos, Nigeria; Tamale, Ghana; and Jos, Nigeria). (b) Functional response curves of host occupancy to human population density for the climate-based occupancy modelling (blue) and the IMSOM derived occupancy modelling (orange). Figure 3. long description.

Spatially, this ecological tolerance manifests as an annular occupancy pattern in large megacities like Lagos. Rather than being restricted to the rural hinterland, the reservoir persists at high densities within informal settlements and peri-urban agricultural fringes (Figure 3; occupancy patterns for all 12 focal cities are provided in Supplementary Figures 2 and 3).

### Predictors of LASV prevalence

**Figure 4.**
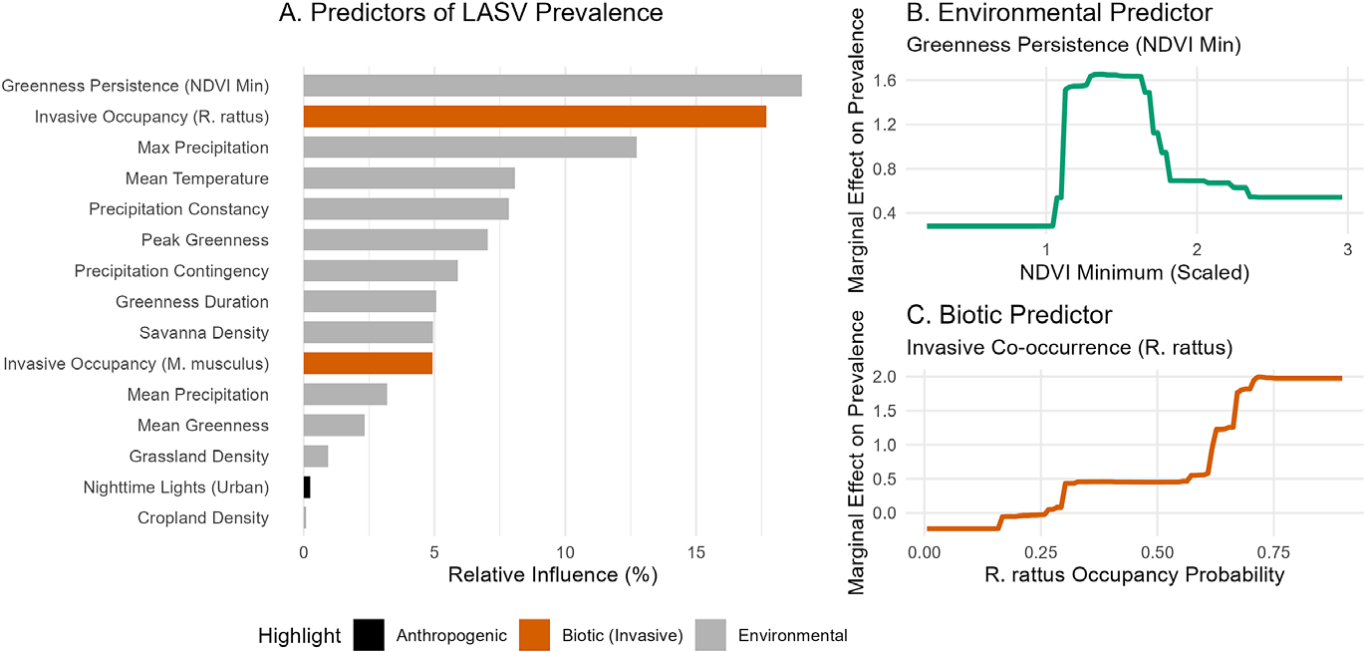
Predictors of Lassa virus prevalence. (a) Variable importance from the BRT model. (b) Partial dependence plot showing the non-linear association with greenness persistence (NDVI minimum). (c) Partial dependence plot showing the positive association with *R. rattus* occupancy. Figure 4. long description.

BRT analysis indicates that LASV virus prevalence within the reservoir is driven by a complex interplay of environmental stability and biotic interactions (Figure 4). While the strongest predictor was greenness persistence (NDVI Min, 19.0%), indicating a preference for habitats with stable vegetation cover – biotic interactions were highly influential. The predicted occupancy of the invasive Black Rat (*R. rattus*) was the second most important variable (17.7%), showing a strong positive association with viral prevalence in *M. natalensis.*

Bivariate partial dependence profiles (Supplementary Figure 4) reveal that peak viral prevalence does not occur in the most environmentally stable habitats (highest NDVI). Instead, it occupies a specific ecological niche characterized by intermediate greenness persistence, indicative of derived savannah or agricultural mosaics, co-occurring with high *R. rattus* occupancy.

**Figure 5.**
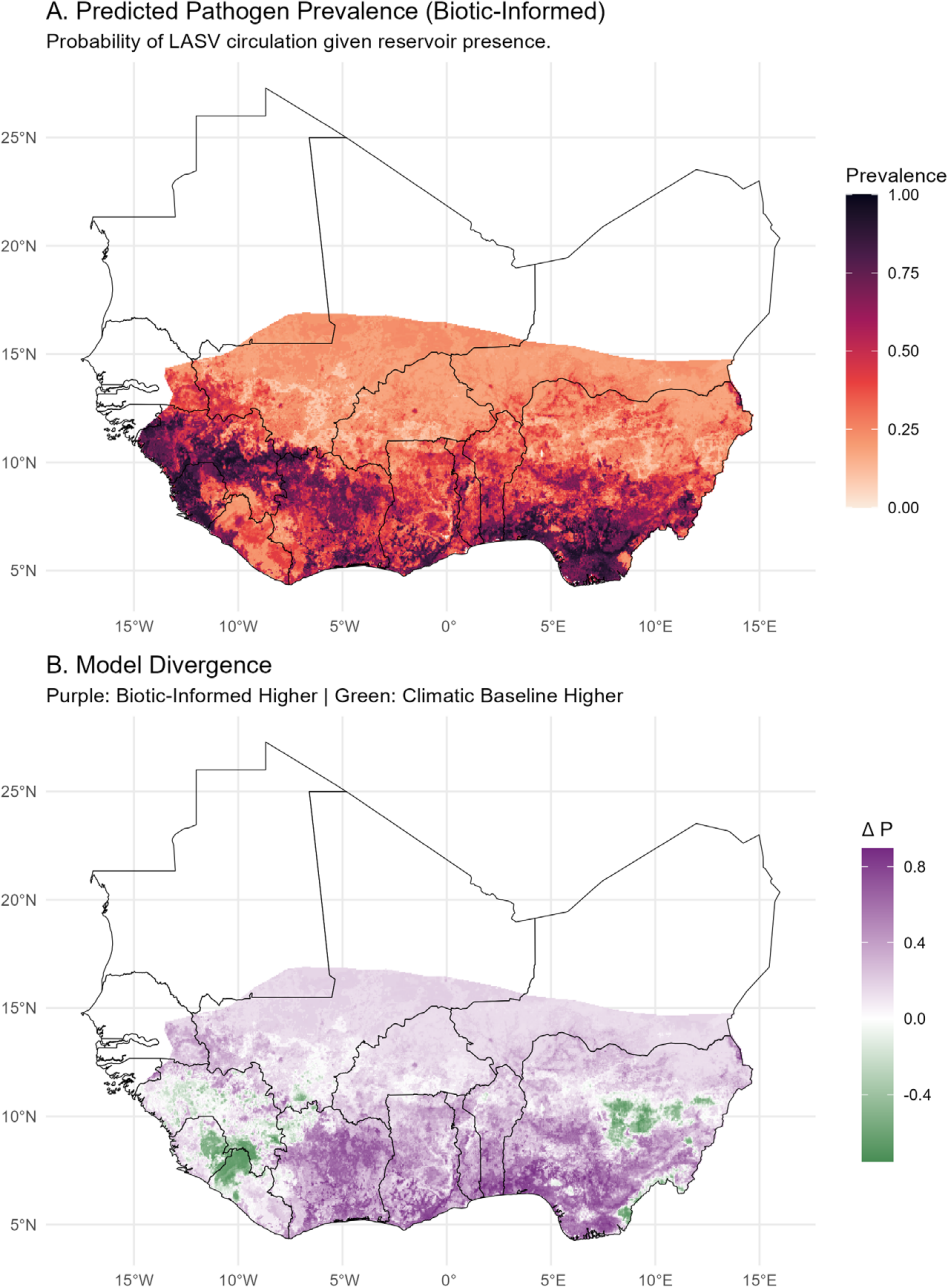
Predicted pathogen hazard ( $D_{L}$ ). Spatial distribution of viral prevalence in the reservoir host, conditional on reservoir presence. Figure 5. long description.

The resulting biotically informed pathogen hazard layer ( $D_{L}$ ) indicates that viral circulation remains high in fragmented, human-dominated landscapes (Figure 5). Predicted viral suitability extends extensively across the derived savannah and peri-urban zones of Nigeria, Benin, and Togo, moving beyond the strict forest-margin zones emphasized in previous analyses.

**Figure 6.**
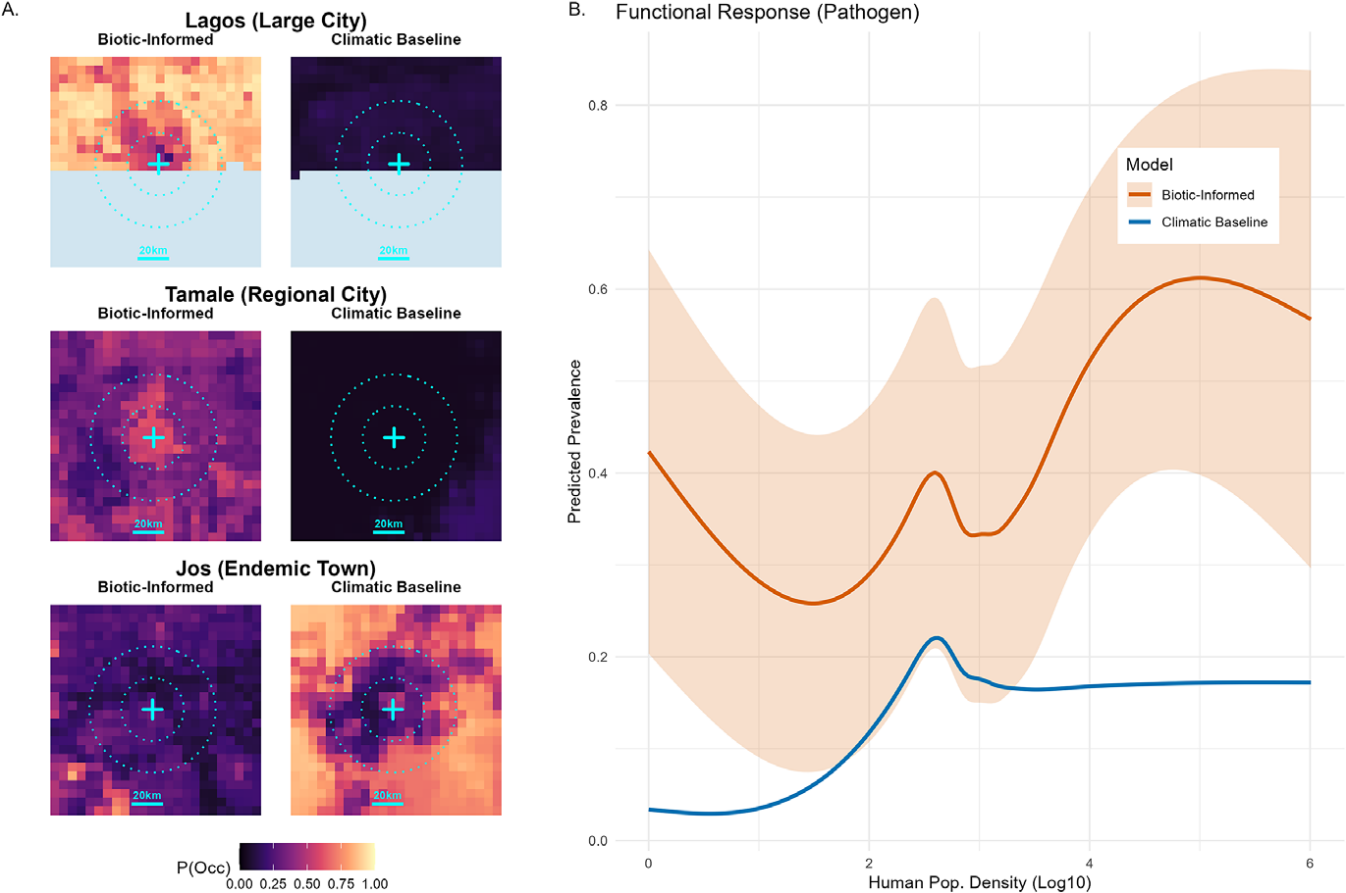
Pathogen urban tolerance. (a) Predicted viral prevalence ( $D_{L}$ ) in and around key cities, contrasting the biotically informed model (left) with the climatic baseline (right). Contours indicate distance from city centre (20 km). (b) Functional response of predicted prevalence to human population density (log10), showing the custom model’s (orange) persistence at higher densities compared to the climatic baseline (blue). Figure 6. long description.

This urban tolerance extends to the pathogen itself. While baseline climatic models predict a strict decline in viral prevalence as human population density increases (peaking at ~2.5 $\log_{10}$ density before flattening), the biotically informed model predicts a resurgence of viral risk at higher densities ( $\log_{10}$ density ~ 4–5). The predicted hazard is maintained in these dense transition zones before finally dropping in the most intensely urbanized commercial cores (Figure 6; urban zooms in Supplementary Figures 4 and 5).

### The socio-economic shield and the burden of infection

To estimate spillover risk, I calculated the composite ecological hazard ( $D_{X}=D_{M}\times D_{L}$ ). However, despite this widespread ecological hazard, human infection patterns do not scale linearly with reservoir presence. I identified a non-linear socio-economic shield effect, where infrastructure quality (proxied by NTL) dampens transmission efficiency.

**Figure 7.**
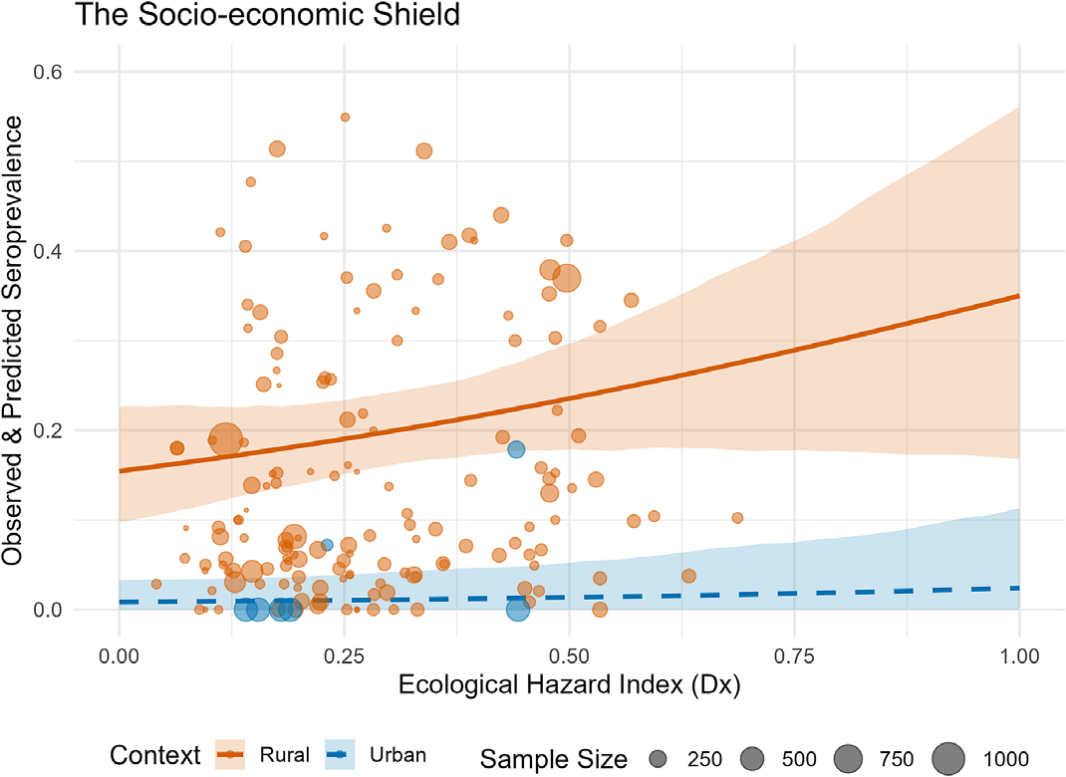
The socio-economic shield. Bootstrapped calibration curves showing the decoupling of ecological hazard and human seroprevalence in urban (blue) versus rural (orange) settings. Ribbons indicate 95% confidence intervals; points indicate raw, unbinned observed seroprevalence from human serosurveys, with point size proportional to the total sample size of each study. Figure 7. long description.

Bootstrapped calibration curves demonstrate that at equivalent levels of ecological hazard ( $D_{X}$ ), human seroprevalence is significantly lower in urbanized settings compared to rural settings. This divergence is driven by a semi-mechanistic calibration: the urban curve is anchored by the limited available empirical urban serosurveys alongside synthetic absence priors representing hyperurban cores, effectively forcing the model to acknowledge the historical absence of transmission in these highly developed zones. This decoupling is visually corroborated by the raw, unbinned data (Figure 7), which demonstrate how these heavily weighted urban anchors pull the expected seroprevalence strictly below the rural baseline.

**Figure 8.**
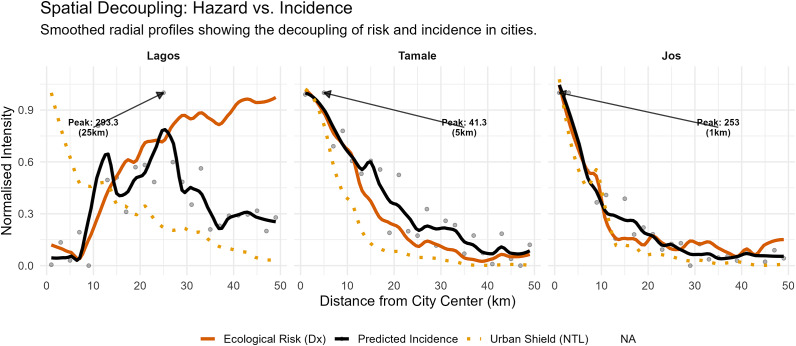
Spatial decoupling of hazard and risk. Radial profiles of ecological hazard (orange), infrastructure shield (dotted yellow), and predicted incidence (black) for representative cities. Lines represent smoothed means (LOESS); arrows indicate the location of peak incidence. Figure 8. long description.

The interplay between this ecological hazard and the socio-economic shield creates distinct transmission typologies characterized by strict spatial decoupling (Figure 8). To quantify this, radial risk profiles were extracted for a stratified sample of 104 West African settlements. Visualization of the full profiles of ecological risk, infection incidence, and NTL are shown in Supplementary Figure 7 for the 12 focal cities.

Urban typology significantly predicted the spatial location of peak incidence (Kruskal-Wallis $\chi^{2}=13.34$ , df = 2, p=0.001 ):

- *Towns and secondary cities*: In smaller urban centres (pop <300000), the shield is effectively absent. Ecological hazard and human incidence overlap spatially, resulting in intense transmission concentrated in the urban core (*n* = 61, median = 1 km, IQR = 1–5 km).
- *Regional cities*: In intermediate cities, a transitional profile is observed. Risk begins to displace from the centre, resulting in a bridge typology between rural towns and major metropolises (*n* = 23, median = 5 km, IQR = 1–7 km).
- *Large cities*: In major metropolitan areas (pop >1 million), the risk profile shifts significantly outward. The median peak incidence is displaced to 8 km from the centre, with a wide variance (*n* = 20, IQR = 3–14 km) that reflects the heterogeneity between unshielded inland cities (e.g. Ibadan) and strongly shielded developed cities (e.g. Lagos).

### The burden of infection and surveillance gaps

**Figure 9.**
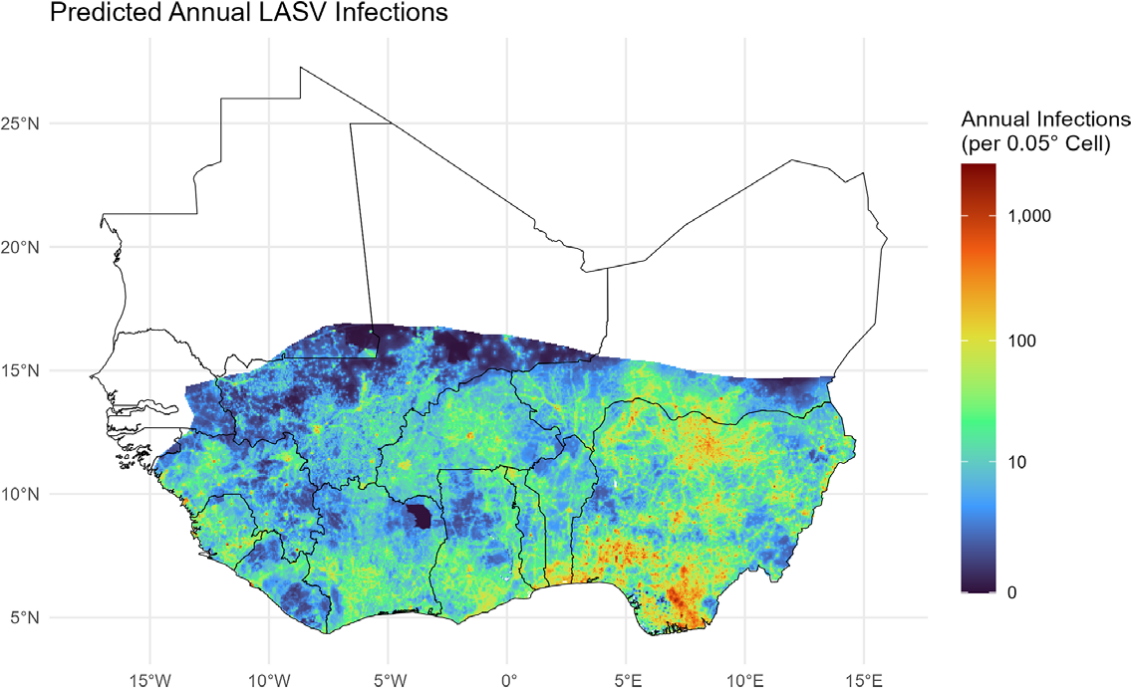
Predicted annual incidence of Lassa virus infection. Estimates account for seroreversion ( λ=0.03 ) and the urban shield effect. Scale is log transformed. Figure 9. long description.

Accounting for this spatial decoupling of hazard and risk yields revised regional burden estimates. Previous models assuming lifelong immunity and rural-dominated transmission estimated approximately 900000 annual infections across the region. By incorporating the urban shield and correcting for seroreversion ( $\lambda =0.03\;{\text{year}}^{-1}$ ), I estimate the annual burden of LASV infection to be approximately 2.6 million infections (sensitivity range: 0.9–4.4 million, depending on immunity duration) (Figure 9).

Notably, while Nigeria bears the highest absolute burden due to its population size (1.5 million annual estimated infections), the per-capita infection rates are highest in the Mano River Union region (Guinea: 7.6 per 1000; Sierra Leone: 7.2 per 1000; and Liberia: 6.1 per 1000) and the silent countries of Benin (7.1 per 1000) and Togo (5.6 per 1000) (Table 1).

**Table 1.** Estimated annual burden of Lassa virus infection by country Table 1. long description.

Country	Infections (thousands)	Rate (per 1000 people)^a^
Guinea	100.2	7.6
Nigeria	1,507.8	7.4
Sierra Leone	61.5	7.2
Benin	88.8	7.1
Niger	163.0	6.7
Burkina Faso	134.9	6.5
Liberia	30.1	6.1
Ivory Coast	153.2	5.8
Togo	46.1	5.6
Mali	110.2	5.4
Ghana	142.9	4.5
Mauritania	4.7	0.9
Senegal	3.2	0.2
Guinea-Bissau	0.1	0.1

Estimates derived from the full custom (biotic + shielded) model assuming a seroreversion rate of λ=0.03 year^a^. Countries are ordered by infection rate (per 1000 people).

a Rate calculated using 2020 WorldPop population estimates constrained to the study region.

**Figure 10.**
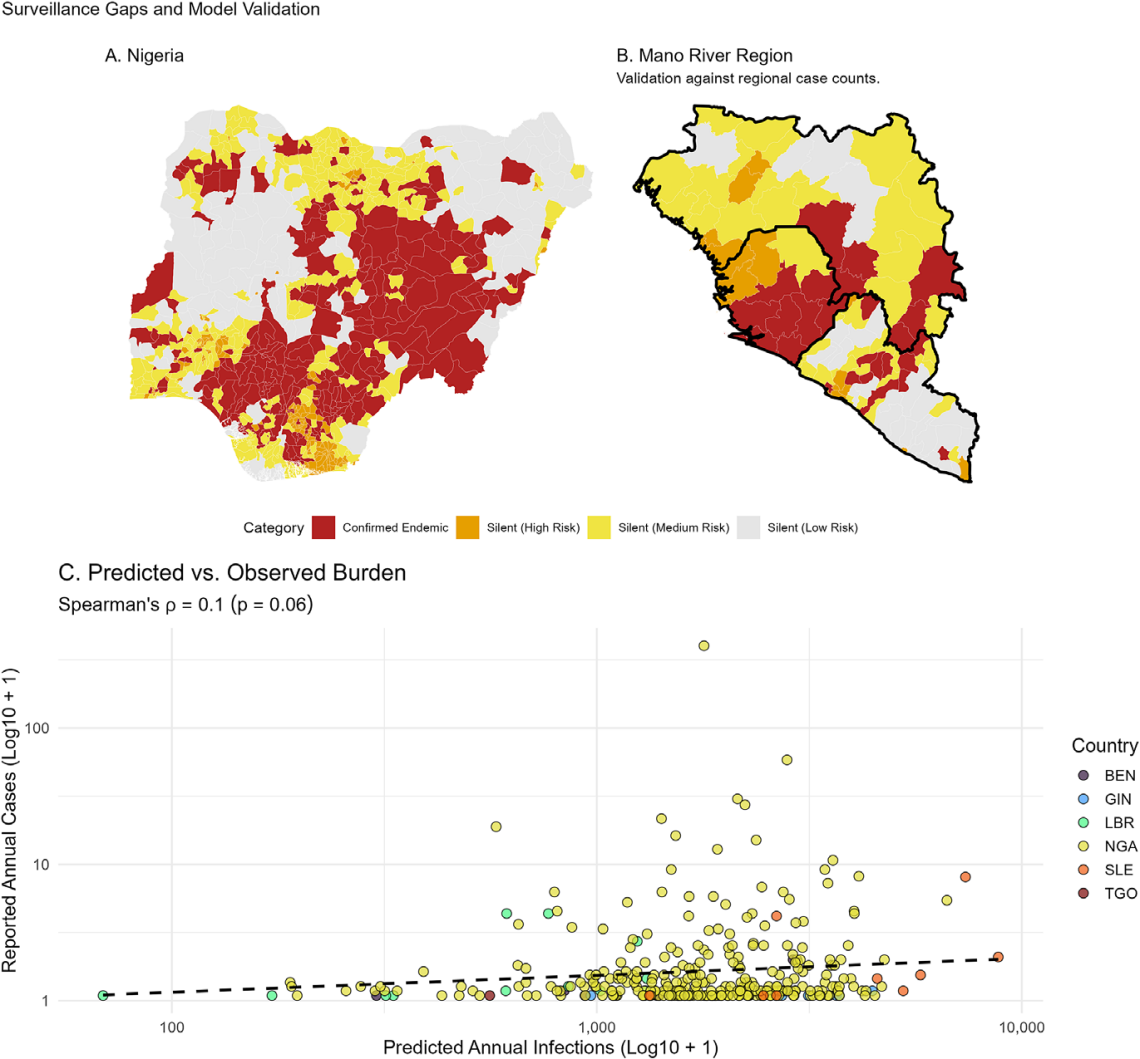
Surveillance gaps and model validation. (a) Nigeria: risk stratification of LGAs with zero reported cases (NCDC data). (b) Mano River Union: validation against historical case counts (Moore et al.). Red districts indicate confirmed endemic presence; orange and yellow districts represent ‘silent’ areas with high predicted risk but zero reported cases. (c) Scatter plot of predicted annual infections versus reported annual cases across all subnational districts, coloured by country, illustrating the impact of zero-reporting on overall correlation. Figure 10. long description.

Finally, comparing predicted annual infections against reported clinical case counts reveals substantial surveillance gaps (Figure 10). Across the entire region, the overall rank correlation between predicted and observed burden is weak (Spearman’s ρ = 0.10, *p* = 0.06). However, geographical stratification demonstrates that this is heavily driven by regional reporting variance. In the Mano River Union, where historical research is robust, predictions align more closely with historical case counts (AUC = 0.66, ρ = 0.24). Conversely, in Nigeria, the correlation degrades (AUC = 0.55, ρ = 0.07) as the model identifies numerous silent districts, areas with top-quartile predicted incidence but zero reported cases.

## Discussion

### Redefining the reservoir niche: The urban blind spot

Previous spatial models have consistently predicted the exclusion of the *M. natalensis* reservoir from heavily human-modified landscapes [8]. By integrating multispecies interactions into an IMSOM framework, I reveal that the reservoir’s realized niche is significantly constrained by the wider rodent community structure, resulting in high predicted suitability in the peri-urban fringes of major West African cities (Figure 2). This urban blind spot in earlier models likely stems from the substantial rural sampling bias identified in historical trapping data (Figure 1), aligning with recent field surveys documenting *M. natalensis* persisting alongside invasive species in human settlements [1, 14, 27].

This urban tolerance extends to the pathogen itself. The current model indicates that LASV hazard is sustained in dense peri-urban transition zones where the reservoir and invasive competitors (*R. rattus*, *M. musculus*) co-occur. While the exact mechanisms of this interaction, whether competitive exclusion or stable co-existence, require finer-scale local study [1, 14], the regional epidemiological implication is clear: active surveillance must encompass these expanding peri-urban agricultural belts.

### The socio-economic shield: A mechanism for non-linear risk

A limitation of prior spatial models is their reliance on linear, density-dependent transmission assumptions, which project biologically implausible contact rates in megacities [8]. The identification of a socio-economic shield (Figure 7) demonstrates that urban infrastructure decouples reservoir presence from human infection. NTL effectively proxies this transition in housing quality and human behaviour [33]. Consequently, shielded city centres remain relatively LASV-free despite high biological hazard, while unshielded peri-urban fringes suffer intense transmission pressure (Figure 8).

### Resolving the burden and silent districts

This biotically informed model estimates approximately 2.6 million annual LASV infections across West Africa (Table 1). This exceeds prior estimates of 300000 to 900000 [2, 8], primarily due to incorporating an empirically modelled seroreversion rate ( $\lambda =0.03\;{\text{year}}^{-1}$ ) rather than assuming lifelong immunity. Notably, previous modelling has estimated up to 4.3 million annual infections when allowing for seroreversion [8]. The more conservative 2.6 million burden estimated here reflects the suppressive effect of the socio-economic shield, which recalibrates the spatial intensity of transmission out of high-density urban cores.

However, this high infection burden does not imply millions of clinical LF cases. Applying the hospital-derived case fatality rate (~18%) would suggest >460000 annual deaths, contradicting regional all-cause mortality data. Instead, this highlights that the vast majority of infections cause asymptomatic or paucisymptomatic seroconversion. The implied IFR is approximately 0.2% to 0.4%, consistent with early historical estimates [2].

This extensive under-reporting is strongly supported by the spatial validation. While predictions align moderately well with clinical data in the relatively well-surveilled Mano River Union, this correlation primarily reflects epidemic surveillance. Epidemic surveillance is inherently reactive and heavily biased towards severe, hospitalized outbreaks. The model identifies numerous silent districts in Nigeria, Benin, and Togo (Figure 10), representing top-quartile incidence areas with zero reported cases. As highlighted by anthropological scholarship, this epidemiological invisibility is actively produced by structural inequalities and diagnostic scarcity, rather than an absence of biological hazard [3, 4].

True endemic surveillance, which would capture the continuous background noise of paucisymptomatic transmission, is rarely conducted for LF. Currently, our understanding of this endemic burden relies almost entirely on targeted academic research, such as the serological datasets from Sierra Leone and Guinea used to calibrate this model. Moving forward, large-scale, multi-country prospective cohorts like the ENABLE study [34] will be critical to resolving these silent districts and shifting the paradigm from reactive outbreak response to proactive endemic mitigation.

### Implications for control and elimination

These distinct urban risk typologies require stratified LF control. In towns where the socio-economic shield is weak, vector control and housing improvements must target the urban core. Conversely, in megacities (e.g. Lagos, Abidjan), resources should be prioritized towards the peri-urban fringe (8–20 km radius) where high reservoir density and unshielded housing collide. Translating these spatial findings into public health action requires moving beyond reactive culling. Indiscriminately targeting all rodents frequently yields diminishing returns and risks competitive release, where the removal of one species can facilitate the rapid population expansion and indoor habitat shift of a competitor [35, 36]. Because *R. rattus* occupancy strongly predicts viral prevalence (Figure 4) but likely signals highly disturbed peridomestic habitats rather than direct LASV transmission, control must be ecologically targeted. Rather than relying solely on rodenticides, interventions should focus on buttressing the socio-economic shield at the household level. Implementing structural rodent proofing, such as sealing eaves, securing domestic food storage, and establishing collective refuse management, could act to physically decouple the domestic interface from the multispecies rodent community [20, 37].

### Limitations

This study is subject to inherent macroecological limitations. Predictions at a $0.05^{\circ }$ resolution cannot resolve fine-scale household heterogeneity. NTL telemetry struggles to differentiate between planned urban infrastructure and adjacent high-density informal settlements, where a lack of electrification (low light) masks the high biological hazard of poor housing quality. Additionally, the blooming effect of satellite-derived NTL may cause bright urban cores to artificially inflate the perceived protective shield in adjacent peri-urban pixels. Furthermore, the socio-economic shield represents a semi-mechanistic constraint rather than a purely empirical derivation. Because historical community serosurveys are almost entirely restricted to rural populations, the urban calibration curve is heavily reliant on informative synthetic priors designed to prevent the extrapolation of rural contact rates into concrete-dominated city centres. While grounded in the historical absence of urban outbreaks, this shield remains a theoretical construct requiring rigorous field validation. Additionally, *M. natalensis* was treated as a single taxonomic unit, though mitochondrial lineages vary in LASV competence [38]. Finally, assuming statistical independence between host ( $D_{M}$ ) and pathogen ( $D_{L}$ ) layers during uncertainty propagation, despite a moderate spatial correlation ( r=0.54 ), means the derived uncertainty bounds should be interpreted as conservative.

Future work must address the critical data gaps exposed by this analysis. Most urgently, systematic human serosurveys must be conducted within the silent districts and across the peri-urban-to-urban gradient to empirically test the socio-economic shield hypothesis. Furthermore, accurate quantification of the asymptomatic fraction via community-based cohorts is essential to refine these burden estimates.

## Conclusion

LF possesses the biological potential to become a peri-urban disease. By moving beyond simple climatic correlations and explicitly modelling the biotic and socio-economic filters of spillover, this study exposes a cryptic burden of infection extending well beyond currently recognized endemic zones. As West Africa urbanizes, the protective shield of infrastructure will likely lag behind the expansion of the reservoir’s commensal niche. Bridging this gap through targeted surveillance in silent districts and robust investment in peri-urban housing quality is a critical public health priority.

## Supporting information

10.1017/S0950268826101824.sm001 Simons supplementary material Simons supplementary material

## Data Availability

The data and code underlying this article are available in the GitHub repository: https://github.com/DidDrog11/lassa-bridging-reanalysis.

## Supplementary material

The supplementary material for this article can be found at http://doi.org/10.1017/S0950268826101824.

## Long descriptions

### Figure 1. Long description

A three-panel map series labeled A, B, and C.

Panel A, Host Community Sampling (8 Species): Shows West Africa with a dashed line indicating the I U C N range of M. natalensis. Orange dots representing the primary reservoir are widely distributed across the southern half of the region. Blue dots for invasive species are concentrated in coastal Northwest areas like Senegal and Guinea. Grey dots for the native community are scattered throughout.

Panel B, L A S V Prevalence Data: Displays country borders for Mauritania, Mali, Niger, Senegal, Gambia, Guinea-Bissau, Guinea, Sierra Leone, Liberia, Ivory Coast, Burkina Faso, Ghana, Togo, Benin, and Nigeria. Red dots for rodent testing are clustered in Sierra Leone, Guinea, and Southern Nigeria. Gold triangles for human serosurveys are located in Sierra Leone, Liberia, and Central Nigeria. Black asterisks mark urban anchors in major coastal cities.

Panel C, Validation: N C D C Confirmed (Red) and Regional Reports: A large map with an inset showing the study area's location in Africa. Nigeria is highlighted with red administrative areas indicating confirmed cases. Other regions are shaded from dark purple to light orange based on a log-transformed scale of cases from 0.5 to 2.0. Specific city labels include Conakry, Freetown, Kenema, N'Zerekore, Bouake, Abidjan, Tamale, Accra, Lagos, Ogbomosho, Ekpoma, and Jos. High case densities are visible in the forest regions of Guinea, Sierra Leone, and Liberia, as well as Southern and Central Nigeria.

Navigate back to Figure 1..

### Figure 2. Long description

A two-panel vertical layout showing geographical data across West Africa from 5 degrees North to 25 degrees North and 15 degrees West to 15 degrees East.

Panel A, titled Predicted Reservoir Niche Biotic I M S O M, displays occupancy probability P Occ. A color scale on the right ranges from 0.00 dark purple to 1.00 light yellow. The map shows high occupancy probability concentrated in the southern coastal regions and central inland areas, with values decreasing toward the North and the Saharan border.

Panel B, titled Model Divergence, shows the difference in probability Delta P. A diverging color scale on the right ranges from negative 0.8 green to positive 0.8 purple, with white at 0.0. Purple indicates Biotic I M S O M is higher, while green indicates Climatic S D M is higher. The map shows purple concentrations in the Southwest coastal regions and scattered urban zones, while green dominates the central and northern interior of the habitable zone.

Navigate back to Figure 2..

### Figure 3. Long description

Panel A, titled Mechanisms of urban tolerance, contains six heatmaps arranged in three rows for Lagos, Tamale, and Jos. Each row compares Biotic I M S O M on the left with Climate S D M on the right. The maps use a color scale for P Occ from dark purple (0.00) to bright yellow (1.00). Each map features a central cyan crosshair and concentric dotted circles indicating a 20 kilometer scale. Lagos shows high occupancy in the North with a coastal cutoff. Tamale and Jos show varying intensities of occupancy concentrated around the central urban core.

Panel B, titled Host Tolerance to Urbanisation, is a line graph. The x-axis represents Log 10 Human Population Density from 0 to 6. The y-axis represents Predicted Occupancy Probability from 0.00 to 0.75.

- The Climate S D M curve (blue) starts at 0.50, dips to a trough of 0.22 at x equals 1.5, rises sharply to a peak of 0.60 at x equals 3.5, and then plateaus around 0.55.

- The Biotic I M S O M curve (orange) starts lower at 0.15, dips slightly, and then shows a steady, near-linear increase, reaching its highest point of approximately 0.78 at x equals 6. This curve is surrounded by a light orange shaded area representing the confidence interval.

Navigate back to Figure 3..

### Figure 4. Long description

Panel A, titled Predictors of L A S V Prevalence, is a horizontal bar chart with the x-axis showing Relative Influence percentage from 0 to 15. The y-axis lists 14 variables. Greenness Persistence N D V I Min has the highest influence at approximately 18 percent. Invasive Occupancy R dot rattus is second at approximately 17 percent. Other variables include Max Precipitation, Mean Temperature, and Precipitation Constancy. Bars are color-coded: grey for Environmental, orange for Biotic Invasive, and black for Anthropogenic.

Panel B, titled Environmental Predictor, is a line graph of Greenness Persistence N D V I Min. The x-axis is N D V I Minimum Scaled from 0 to 3. The y-axis is Marginal Effect on Prevalence from 0.4 to 1.6. The green line remains low until x equals 1, then sharply peaks at 1.6 between x equals 1.2 and 1.6, before dropping and plateauing at 0.6.

Panel C, titled Biotic Predictor, is a line graph of Invasive Co-occurrence R dot rattus. The x-axis is R dot rattus Occupancy Probability from 0.00 to 0.75. The y-axis is Marginal Effect on Prevalence from 0.0 to 2.0. The orange line shows a stepped positive association, starting at 0.0 and rising sharply after x equals 0.5 to reach a plateau at 2.0.

Navigate back to Figure 4..

### Figure 5. Long description

Two stacked geographical maps of West Africa with a latitude range from 5 degrees North to 25 degrees North and a longitude range from 15 degrees West to 15 degrees East.

Panel A, titled Predicted Pathogen Prevalence Biotic-Informed, displays the probability of L A S V circulation. A color scale to the right ranges from 0.00 in light peach to 1.00 in dark purple. The highest prevalence, indicated by dark purple and deep red, is concentrated in the Southwest coastal regions, specifically around Sierra Leone, Liberia, and Guinea. The prevalence decreases to light orange as it moves North toward the 15 degrees North latitude line.

Panel B, titled Model Divergence, shows the difference in probability, delta P. The legend indicates purple for Biotic-Informed Higher and green for Climatic Baseline Higher, ranging from negative 0.4 to 0.8. Most of the southern region is shaded in light to medium purple, indicating the biotic-informed model predicts higher prevalence. Small, distinct pockets of green are visible in the Southwest and Southeast, where the climatic baseline model predicts higher prevalence. The central and northern parts of the mapped area appear in light purple or white, indicating low divergence.

Navigate back to Figure 5..

### Figure 6. Long description

Panel A contains six heatmaps arranged in three rows for Lagos, Tamale, and Jos. Each row compares a Biotic-Informed model on the left to a Climatic Baseline on the right. Concentric dotted circles indicate 20 km intervals from the city center marked by a cyan cross. In Lagos and Tamale, the Biotic-Informed maps show high prevalence (yellow/orange) concentrated at the center, while the Climatic Baseline shows low prevalence (dark purple). In Jos, the pattern is reversed. A color scale at the bottom indicates P(Occ) from 0.00 (dark) to 1.00 (light yellow).

Panel B is a line graph showing Predicted Prevalence on the Y-axis (0.0 to 0.8) against Human Pop. Density (Log 10) on the X-axis (0 to 6). The Biotic-Informed model (orange line) starts at 0.4, dips, peaks at a density of 2.5, and then rises to a plateau near 0.6. The Climatic Baseline (blue line) starts near 0.0, peaks at 0.2 at a density of 2.5, and remains flat at 0.2 for higher densities. Shaded areas represent confidence intervals.

Navigate back to Figure 6..

### Figure 7. Long description

The x-axis represents the Ecological Hazard Index (D x) ranging from 0.00 to 1.00. The y-axis represents Observed and Predicted Seroprevalence ranging from 0.0 to 0.6.

Two distinct data series are shown:

* Rural (orange): A solid line showing a positive, slightly exponential increase in predicted seroprevalence as ecological hazard increases. It starts near 0.15 at the y-intercept and rises toward 0.35. A wide orange shaded ribbon represents the 95 percent confidence interval. Numerous orange circles of varying sizes are scattered primarily between 0.0 and 0.5 on the y-axis.

* Urban (blue): A dashed line that remains nearly flat and asymptotic near the 0.0 mark on the y-axis, indicating a decoupling from the hazard index. A narrow blue shaded ribbon represents the 95 percent confidence interval. A small number of blue circles are clustered very low on the y-axis, mostly below 0.1.

A legend at the bottom indicates that point size is proportional to Sample Size, with reference circles for 250, 500, 750, and 1000.

Navigate back to Figure 7..

### Figure 8. Long description

A multi-panel line graph with three horizontal panels. The y-axis represents Normalised Intensity from 0.0 to 0.9. The x-axis represents Distance from City Center in k m from 0 to 50. Three data series are shown: a solid orange line for Ecological Risk D x, a solid black line for Predicted Incidence, and a dotted yellow line for Urban Shield N T L.

* Lagos panel: The Urban Shield starts high at 1.0 and drops rapidly. Ecological Risk and Predicted Incidence start near 0, rising sharply after 10 k m. Predicted Incidence peaks at 25 k m with a value of 293.3, indicated by an arrow.

* Tamale panel: All three metrics start high near the city center. Urban Shield and Ecological Risk decay rapidly toward 0 by 30 k m. Predicted Incidence decays more slowly, peaking at 5 k m with a value of 41.3.

* Jos panel: All three metrics show a steep, near-identical decay from the center. Predicted Incidence peaks at 1 k m with a value of 253 and remains slightly above the other two metrics as they approach 0 beyond 20 k m.

In all panels, grey dots represent raw data points behind the smoothed L O E S S curves.

Navigate back to Figure 8..

### Figure 9. Long description

A geographical heat map titled Predicted Annual L A S V Infections. The Y-axis represents latitude from 5 degrees North to 25 degrees North. The X-axis represents longitude from 15 degrees West to 15 degrees East. A color scale on the right indicates annual infections per 0.05 degree cell, ranging from 0 in dark purple to 1,000 in deep red on a log-transformed scale.

* The highest predicted infection rates, indicated by orange and red clusters, are concentrated in the South and Southeast, specifically in coastal regions around 5 to 10 degrees North and 5 to 10 degrees East.

* Moderate infection rates, shown in green and yellow, span the central belt of the mapped area between 7 degrees North and 15 degrees North.

* Low infection rates, shown in dark blue and purple, are found at the northernmost edge of the colored zone, roughly along the 15 degrees North latitude line.

* The northern half of the map, above 17 degrees North, is white, indicating areas outside the prediction model or with zero expected incidence.

Navigate back to Figure 9..

### Table 1. Long description

The table consists of three columns: Country, Infections in thousands, and Rate per 1000 people. The data is ordered by descending rate:

* Guinea: 100.2 thousand infections, 7.6 rate.

* Nigeria: 1,507.8 thousand infections, 7.4 rate.

* Sierra Leone: 61.5 thousand infections, 7.2 rate.

* Benin: 88.8 thousand infections, 7.1 rate.

* Niger: 163.0 thousand infections, 6.7 rate.

* Burkina Faso: 134.9 thousand infections, 6.5 rate.

* Liberia: 30.1 thousand infections, 6.1 rate.

* Ivory Coast: 153.2 thousand infections, 5.8 rate.

* Togo: 46.1 thousand infections, 5.6 rate.

* Mali: 110.2 thousand infections, 5.4 rate.

* Ghana: 142.9 thousand infections, 4.5 rate.

* Mauritania: 4.7 thousand infections, 0.9 rate.

* Senegal: 3.2 thousand infections, 0.2 rate.

* Guinea-Bissau: 0.1 thousand infections, 0.1 rate.

Footnotes indicate estimates assume a seroreversion rate of lambda = 0.03 per year and use 2020 WorldPop population data.

Navigate back to Table 1..

### Figure 10. Long description

Panel A shows a map of Nigeria divided into L G A districts. A large cluster of dark red Confirmed Endemic districts covers the central and southeastern regions. Orange Silent High Risk and yellow Silent Medium Risk districts are scattered primarily in the North and Southwest. Light gray indicates Silent Low Risk.

Panel B shows the Mano River Region including Guinea, Sierra Leone, and Liberia. Dark red districts are concentrated in the central forest region spanning the borders. Orange and yellow districts surround these endemic areas, particularly to the North and West.

A shared legend between the maps defines four categories. Dark red for Confirmed Endemic. Orange for Silent High Risk. Yellow for Silent Medium Risk. Light gray for Silent Low Risk.

Panel C is a scatter plot titled Predicted versus Observed Burden. The x-axis represents Predicted Annual Infections Log 10 plus 1 on a scale from 100 to 10,000. The y-axis represents Reported Annual Cases Log 10 plus 1 on a scale from 1 to 100. Data points are color-coded by country. B E N in dark purple. G I N in blue. L B R in green. N G A in yellow. S L E in orange. T G O in red. A dense horizontal cluster of points sits along the y-axis value of 1, indicating many districts with high predicted risk but zero reported cases. A dashed black trend line shows a very slight positive slope. Spearman's rho equals 0.1 with a p-value of 0.06.

Navigate back to Figure 10..
